# Extraction of Light Using Random Nanocone on Poly(vinyl-butyral) for Flexible OLEDs

**DOI:** 10.1038/s41598-019-48808-8

**Published:** 2019-08-23

**Authors:** Dong Jun Lee, In Seon Yoon, Cheol Hwee Park, Junhee Choi, Young Wook Park, Byeong-Kwon Ju

**Affiliations:** 1 Display and Nanosystem Laboratory, Department of Electrical Engineering, Korea University, 145, Anam-ro, Seongbuk-gu, Seoul, 02841 Republic of Korea; 2 School of Mechanical and ICT Convergence Engineering, SUN MOON University, Chungcheongnam-do, 31460 Republic of Korea

**Keywords:** Electrical and electronic engineering, Nanowires, Nanowires

## Abstract

In this study, we designed a smooth, highly flexible, mechanically robust poly(vinyl-butyral) (PVB)/silver nanowire (AgNW) composite transparent conducting electrode (TCE) integrated with a random nanocone (RNC) to enhance the light extraction of flexible organic light-emitting diodes (OLEDs). The RNC was fabricated by reactive-ion etching (RIE) on AgNW embedded in PVB. As the etching time increased, the size of the RNC became larger. The sheet resistance and transmittance of PVB/AgNW with the RNC was 21.7 Ω/sq and ~87%, respectively. For the PVB/AgNW, the change in sheet resistance was only 2.6% when a 2,000-bend test was performed. The maximum external quantum efficiency was 28.3% when RNC 700 s was used as a green phosphorescent OLED. In addition, for current efficiency and power efficiency, RNC 700 s increased 1.4 times over RNC 0 s. RNC is free of viewing-angle-dependent color and brightness distortion. PVB/AgNW and RNC are practical ways to overcome the brittleness of conventional indium tin oxide and improve the efficiency of flexible OLEDs. Finally, this product is expected to be applied to various flexible optical devices.

## Introduction

It is very important to produce a transparent electrode film on a flexible substrate. This is because it is essential for realizing future optoelectronic devices such as touch panels, organic photovoltaic cells, and organic light-emitting diodes (OLEDs)^1^. Although the continuous development of optoelectronic devices has been undertaken over the past two decades, the development of flexible electronic devices is still lagging owing to the absence of ideal flexible transparent electrodes requiring high electrical conductivity, optical transparency, and mechanical flexibility. In the case of flexible electronic devices, indium tin oxide (ITO) electrodes, the most commonly used transparent electrodes, cannot be applied because they are easily broken^2^.

To overcome these shortcomings, various flexible transparent conductive materials such as metal nanowires^3–5^, conductive polymers^6–9^, ultrathin metal films^10,11^, carbon materials^12,13^, polymer metals^14^, and some hybrids such as graphene/conductive polymers^15^ have been studied. However, all of these electrodes have serious problems such as low stability, noticeable color of conducting polymers^16,17^, high surface roughness, and a high corrosion rate of metal nanowires^18^. In addition, there is a trade-off between the high sheet resistance (R_s_) of the carbon material and the transmittance of the ultrathin metal film. Among these competing electrodes, AgNW-based transparent conducting electrodes (TCEs) exhibit competitive optoelectronic performance compared to ITO^19,20^. Nevertheless, the weak adhesion of AgNW to the substrate surface and its high surface roughness are not suitable for high-efficiency flexible OLEDs. Therefore, embedding the AgNW into the substrate is considered a promising solution. A recent research proposed coating the AgNW on a flat glass and embedding it on other flexible substrates. These electrodes exhibited extremely low roughness, low R_s_, and high transmittance. Due to such superior performance, the embedded AgNW was used in various applications, such as resistive random-access memory^21^, electroluminescence devices^22,23^, and OLEDs in our former research^24^.

In addition to the aforementioned issues, most of the generated light has been reduced by ~30% in planar structures owing to waveguides (WG), substrate modes, and surface plasmon polaritons (SPP) even though the internal quantum efficiency of OLED emissive materials has increased by up to 100%^25,26^. We embedded AgNW in poly(vinyl-butyral) (PVB) to overcome the low adhesion and high roughness of the AgNW. PVB has flexibility, strong adhesion, optical transparency, and inexpensive properties^27^. Therefore, by embedding AgNW into PVB easily at room temperature, the surface roughness was reduced, the adhesion to the substrate was increased, and a flexible electrode with excellent optical properties was fabricated.

To overcome the limited optical outcoupling efficiency, various strategies of internal and external light extraction methods have been studied in the field of OLEDs. Internal light extraction uses various nano and micro patterns between the substrate and the device to cause light scattering to overcome the WG or SPP mode^28–31^. However, the electrical characteristics may deteriorate when a pattern is inserted between the substrate and the device for internal light extraction. In the external light extraction method, the electrical characteristics can be preserved and the substrate mode is removed by attaching a microlens array to the substrate^32–35^. However, the conventional manufacturing process requires additional steps to attach the microlens or additional molds. Plasma-induced surface texturing of plastics has been adopted to overcome this problem. Peng *et al*. used etched nanosphere arrays as a mask for Ag evaporation^36^, and Kwack *et al*. constructed random nanoscale rods by etching a photoresist using the reactive-ion etching (RIE) technique^37^. In both these studies, the light extraction efficiency of OLED improved, thereby preserving the electrical properties. As a part of these things, we fabricated a random nanocone (RNC) on PVB through RIE etching without additional mold or electrical degradation^37^. The fabricated RNC can be randomly configured and scaled by the etching time.

This paper reports on data for improving the output coupling efficiency in OLEDs produced by applying various types of RNCs to PVB/AgNW. The RNCs were constructed on the opposite side of the PVB/AgNW substrate for enhancing the light outcoupling of OLEDs. The RNC can be obtained using the run time of the accelerated ions without additional masks for etching, controlled by the plasma condition. Atomic force microscopy (AFM) and scanning electron microscopy (SEM) measurements were performed to investigate the film surface. We performed current density-voltage-luminance (J-V-L) measurements to investigate the electrical and optical properties of OLEDs according to the etching time of the RNCs. A viewing angle analysis was performed to evaluate the OLED light extraction.

## Results and Discussion

**Figure 1:**
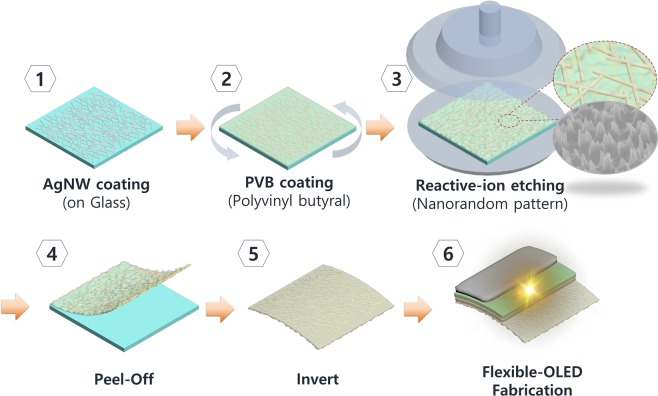
Fabrication procedure of random nanocone (RNC) and PVB/AgNW transparent electrodes.

The fabrication process of the RNC flexible transparent electrode for a light extraction layer is shown in Fig. 1. The glass (Eagle XG, Corning Co., Ltd.) is immersed in acetone, methanol, and deionized water and washed for 15 min in each by ultrasonication. Thereafter, nitrogen gas is blown to dry the glass. The dispersed AgNW is spin-coated (500 rpm for 20 s) on the glass and the organic solvent residue is evaporated by heat treatment at 60 °C for 1 min. The PVB solution is prepared to fabricate a flexible substrate and is then spin-coated on the AgNW. RIE was performed using plasma sulfur hexafluoride (SF_6_) and oxygen (O_2_) plasma to form an RNC. The oxygen ratio of the gas mixture is known to affect various factors including the anisotropy, etch rate, and shape of etched pattern^38–40^. We chose a 10:1 ratio to render the nanocone shape with random position, which is advantageous during light extraction^40^. Nanocone shape can induce the refractive graded-index effect that can suppress the reflection of the interfaces between two different media^41^. The randomly patterned PVB is soaked in water and peeled from the glass. The poly(3,4-ethylenedioxythiophene)-poly(styrenesulfonate) (PEDOT:PSS) solution was spin-coated on AgNW at 2000 rpm to planarize the surface.

**Figure 2:**
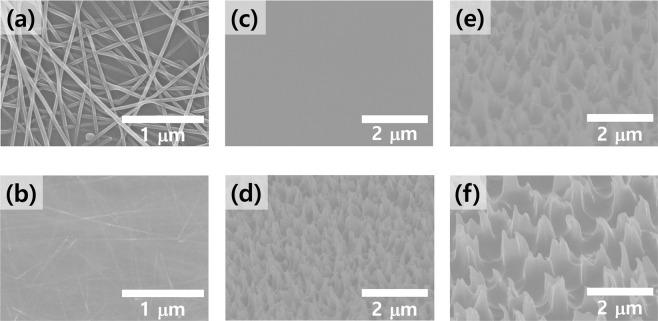
SEM images of (**a**) AgNW networks formed on glass and (**b**) AgNW networks embedded in PVB. SEM images of various PVB/AgNW on glass substrates formed by SF_6_ and O_2_ plasma treatment. Duration of SF_6_/O_2_ plasma treatment was altered to create different RNCs: (**c**) 0 s, (**d**) 300 s, (**e**) 500 s, and (**f**) 700 s. Conditions of the other processes remained unchanged.

SEM images of AgNW coating on glass substrate are shown in Fig. 2(a), and the AgNW embedded PVB electrode is shown in Fig. 2(b). A root-mean-square surface roughness (RMS) of 8 nm and maximum peak-to-valley (R_pv_) range of 77.0 nm were noted for the AgNW layer on glass [Fig. S1(a)]. The protruding nanowires caused local height elevation and increased the possibility of electrical shorting of the device. By contrast, the AgNW-embedded PVB electrode exhibits a remarkably reduced surface roughness with RMS of 0.17 nm and R_pv_ of 2.26 nm, as shown in Fig. 2(b) and Supplementary Fig. S1(b). This highly flat surface morphology implies that the spin-coated PVB liquid polymer well soaked the AgNW network and filled the holes in the networks and voids at the AgNW and rigid substrate interface. Figure 2(c–f) shows the RNC surface morphology when RIE was performed on the PVB.

To maximize the outcoupling of light, choosing a nanoscale structure and size similar to the wavelength of the light is a promising method. The surface topography of the RNC was measured using AFM (Fig. S2) and SEM. Figure 2(c–f) shows the SEM images of the fabricated RNCs with various plasma treatment durations. The SF_6_/O_2_ plasma treatment durations were varied for 0, 300, 500, and 700 s, while the other conditions remained unchanged. The images indicate that increasing the plasma treatment duration results in the increase in the height and width of the RNC structure. The RMS roughness becomes saturated at a higher plasma dose because of the offsetting between etching and reformation.

**Figure 3:**
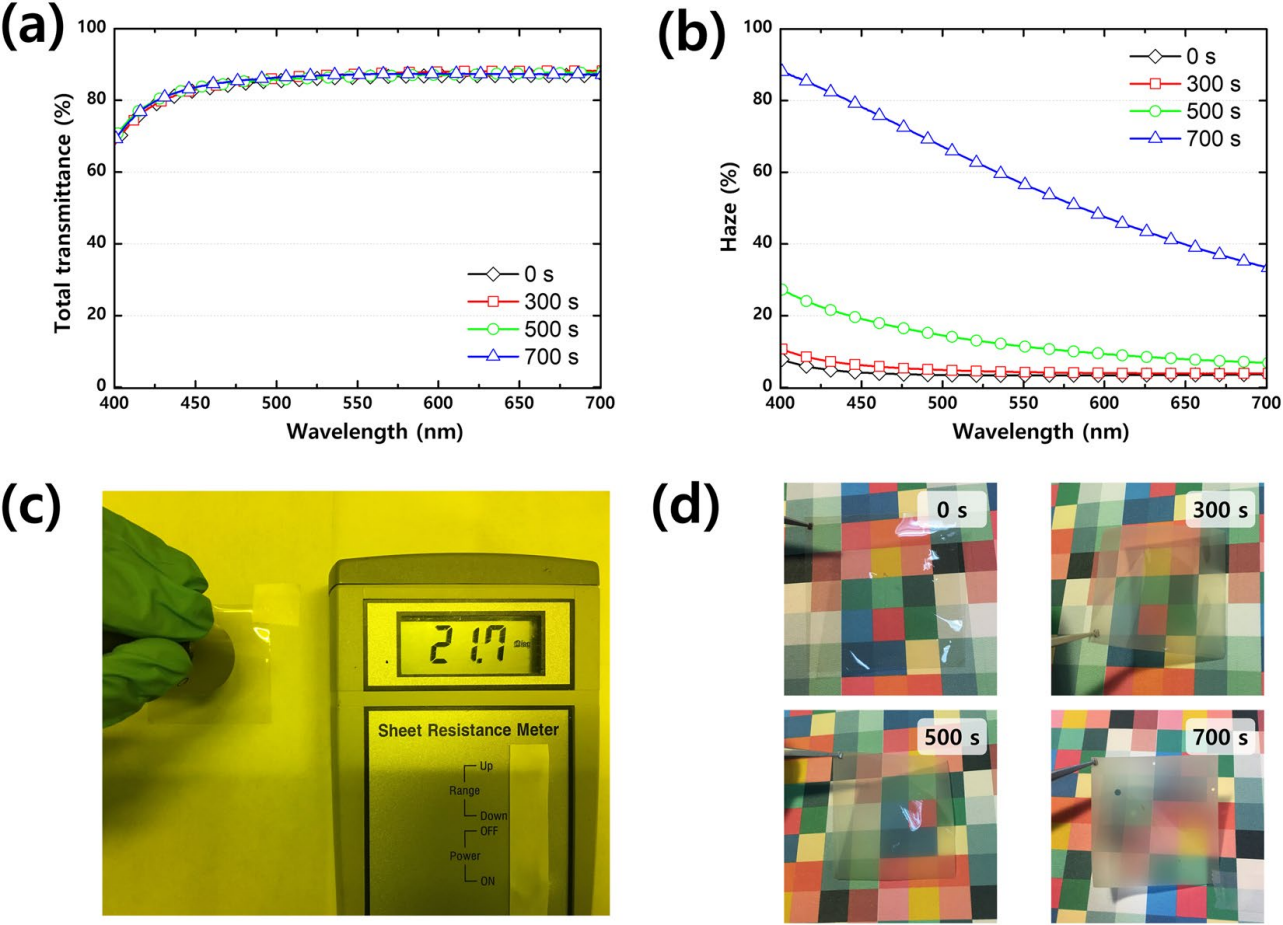
(**a**) Total transmittance and (**b**) haze as a function of wavelength for plasma-treated PVB/AgNW substrates (process pressure: 30 mTorr, process power: 200 W). (**c**) Sheet resistance testing of PVB/AgNW using sheet resistance meter. (**d**) Photographic images of plasma-treated PVB/AgNW films for different plasma times.

Total transmittance of PVB/AgNW for different etching times are shown in Fig. 3(a). The average value of the total transmittance (T_avg_) over the visible-light wavelength range (400–700 nm) was measured as 85.3% in untreated PVB/AgNW, but there was no significant change in transmittance as the etching time increased (T_avg_ = 85.8–86.2%) [Fig. 3(a)].

1 $${\rm{Haze}}=\frac{{\rm{Diffuse}}\,{\rm{transmission}}}{{\rm{Total}}\,{\rm{transmission}}}$$

In contrast, the haze increased progressively as the etching time increased in the visible wavelength region [Fig. 3(b)]. At RNC 0 s, the haze was 1.35% (at 550 nm) in the visible wavelength range, but increased gradually to about 80% at RNC 700 s. This phenomenon is owing to the increase in height and width of the nanostructure, which enhances the scattering of incident photons. As shown in Fig. 3(c), the sheet resistance of PVB/AgNW is 21.7 Ω/sq. Although the sheet resistance is higher than that of commercial ITO/glass, it is sufficient for use as a transparent electrode. In addition, the sheet resistance can be adjusted by the concentration of AgNW. Figure 3(d) is a photograph of actual PVB/AgNW film as the etch time increases. Plasma-treated PVB/AgNW films show a milky image through diffuse scattering as the etching time increases.

**Figure 4:**
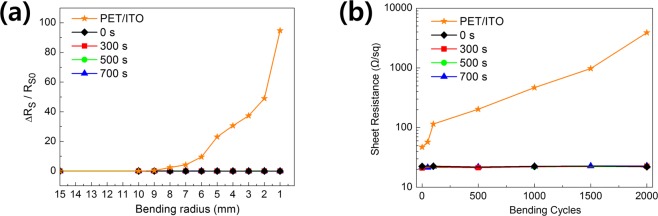
(**a**) Resistance variation of ITO on PET, and AgNW-embedded PVB as a function of bending radius. (**b**) Resistance variation during cyclic test of ITO on PET, and AgNW embedded PVB at a bending radius of 5 mm.

We performed bending tests on PVB/AgNW and PET/ITO, as shown in Fig. 4. PVB/AgNW with etch times of 0, 300, 500, and 700 s was used. ITO films sputtered from PET were evaluated for comparisons in bending tests. PVB/AgNW exhibited an almost constant sheet resistance (R_s_) as the bending radius (R_b_) decreased from 15 to 1 mm. Meanwhile, R_s_ of the ITO film increased significantly at R_b_ = 6 mm. No resistance was observed for R_b_ <2 mm [Fig. 4(a)]. The ITO/PET film began to break during the first few cycles of bending and unfolding, resulting in a sudden increase in the sheet resistance (Fig. 4(b)). The radius of curvature was 5 mm. The reason for this is that ITO is an inorganic material, so it is essentially broken and cracked under minimal strain. However, PVB/AgNW is inherently flexible owing to its integrated and symmetrical form. Accordingly, the sheet resistance of PVB/AgNW does not change after more than 2,000 bending cycles.

**Figure 5:**
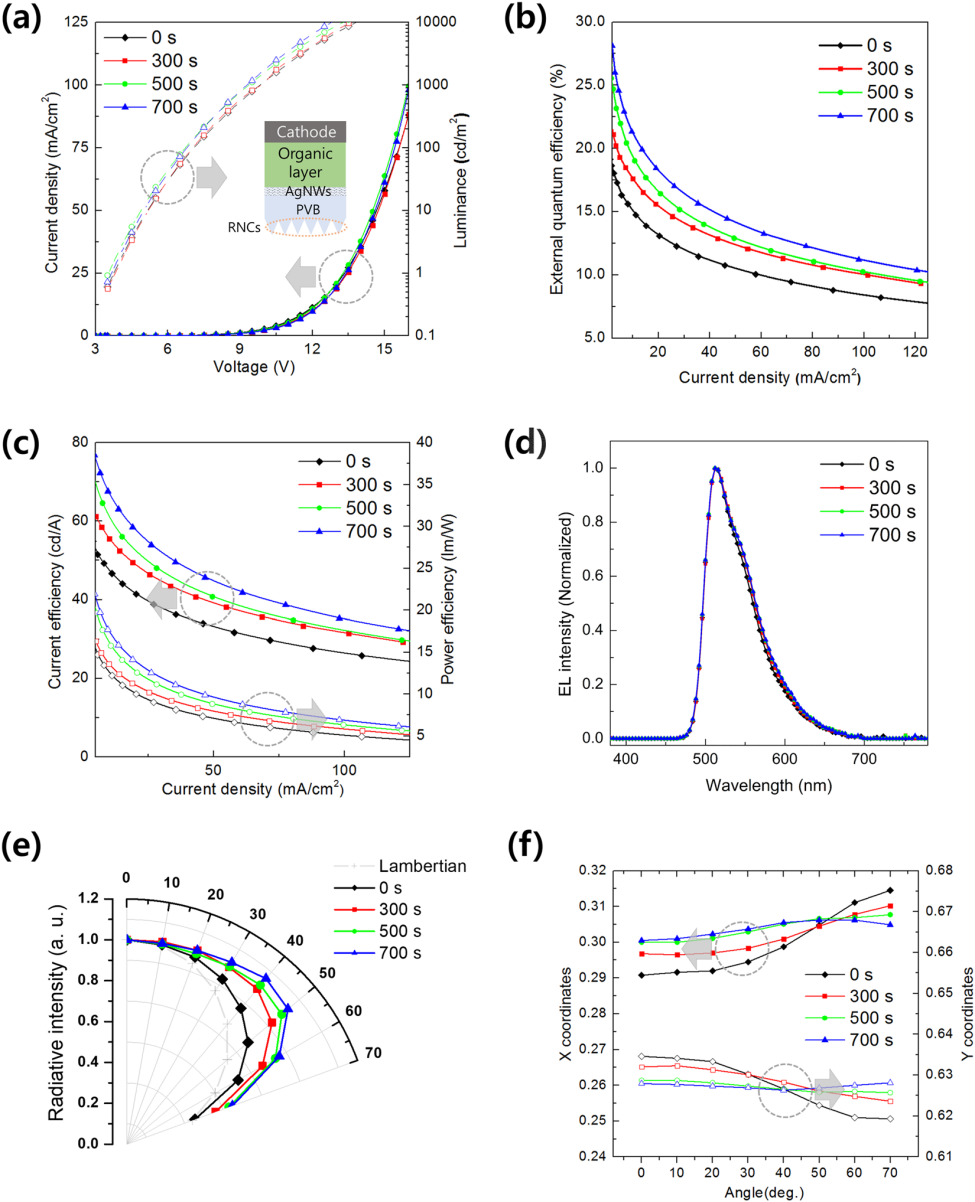
Performance characteristics of phosphorescent green flexible OLEDs: (**a**) current density (J)-voltage (V)-luminance (L) characteristics of OLEDs as a function of etching time, (**b**) EQE as a function of current density, (**c**) CE and PE as a function of current density, (**d**) normalized EL spectra at 1.2 mA/cm^2^, (**e**) normalized angle dependence of electroluminescence intensity (Lambertian emission pattern is displayed as dashed line), and (**f**) CIE X- and Y- coordinates of green OLEDs measured at viewing angles from 0° (substrate normal) to 70°.

Figure 5 shows the characteristics of the fabricated phosphorescent green flexible OLED device. Figure 5(a) shows the J-V-L characteristics of the OLED with the etching time of the RNCs located on the opposite side of the PVB/AgNW. In the J-V curve, there is no significant change when the etch time of the RNC increases. This indicates that the etching process time does not damage the device at all^42,43^. The L-V curves indicate that the device with RNC 700 s has a higher luminance at the same voltage compared to that with RNC 0 s. The external quantum efficiency (EQE) of various green OLEDs are plotted as a function of current density in Fig. 5(b). The corresponding characteristics of various devices are compared and summarized in Table 1. It is obvious that the EQE values of green OLEDs with RNCs were significantly enhanced, and in particular, the use of 700 s resulted in the largest improvement in efficiency. The green OLED with 700 s yielded an EQE of 17.5% at a current density of 25 mA/cm2, which is ≈1.4 times that of the control device (0 s, EQE = 12.5%). The maximum EQE of the green OLED with 700 s was increased to 28.3%. This feature is in good agreement with the increasing trend in the haze for various substrates observed in Fig. 3(b).

**Table 1:** Performance characteristics of phosphorescent green flexible OLED light extraction nanostructures.

Device structures	EQE [25 mA/cm^2^] [%]	R_EQE_	PE [25 mA/cm^2^] [lm/W]	EQE [max] [%]	PE [max] [lm/W]
RNC (0 s)	12.5	1	41.5	19.2	53.4
RNC (300 s)	14.7	1.17	48.8	21.5	62.5
RNC (500 s)	15.5	1.24	52.1	25.7	69.6
RNC (700 s)	17.5	1.4	57.8	28.3	77.1

EQE and PE are compared at their maximum values and at current density of 25 mA/cm^2^. R_EQE_ values are enhancement ratios relative to that of RNC 0 s device at current density of 25 mA/cm^2^.

The current efficiency (CE) and power efficiency (PE) characteristics according to the current density change are shown in Fig. 5(c). As the etching time increases, the power and current efficiency increase the OLED’s efficiency. For instance, the CE and PE of green flexible OLEDs using RNC 700 s at a current density of 25 mA/cm^2^ increased to 57.8 cd/A and 12.9 lm/W, respectively, which are more than 1.4 times higher than those of RNC 0 s (i.e., 41.5 cd/A and 9.26 lm/W). The reason for this enhancement is that there is no optical loss owing to the substrate mode. In particular, the maximum CE and PE values for RNC 700 s were enhanced from RNC 0 s to 16.3 cd/A and 3.6 lm/W, respectively. More important, as the etching time increases, the normalized electroluminescence (EL) spectrum of the green OLED [Fig. 5(d)] does not exhibit any particular wavelength dependence over the entire visible wavelength range. The wavelength of the peak EL intensity for all four devices is 514 nm, and the full width at half maximum (FWHM) is also the same as that for 65 nm in all four devices. The overall tendency of the normalized EL intensity across the entire visible spectrum is similar for all of the devices. Therefore, even when the etching time is changed, there is no significant change in the emission spectrum. The angular dependence of the EL intensity of the device with the etching time of the RNC is plotted in Fig. 5(e). It is noteworthy that using the RNC 700 s in OLEDs results in stronger side emissions compared to the light emissions of RNC 0 s. This is consistent with the increasing characteristics of the haze, as shown in Fig. 3(b).

Figure 5(f) shows the changes in the Commission Internationale d’Eclairage (CIE) coordinates of the green OLED based on the viewing angle according to the etching time. As the etching time increases, the color coordinate changes stabilize. RNC 700 s showed the least change with an increasing viewing angle.

We demonstrated a highly flexible OLED based on a PVB/AgNW electrode and RNC. By embedding AgNW in the PVB, the surface roughness was reduced and the adhesion with the substrate was improved. High transmittance and low sheet resistance make this product suitable for use as transparent electrodes. A bending stability test confirmed that the PVB/AgNW electrode has major advantages over a pristine PET/ITO electrode, especially in terms of mechanical flexibility and durability, thus making it a better candidate for the fabrication of flexible OLEDs. This experimental data shows that RNC 700 s integration increases light extraction efficiency 1.4 times more than RNC 0 s. In particular, the color stability of RNC 700 s was negligible as the viewing angle increased. Therefore, we believe that the aforementioned technologies will contribute to the OLED industry.

## Methods

### Materials

Polyvinyl butyral (PVB, Butvar B-98, Mn = ∼36,000 g/mol) with hydroxyl content in polyvinyl alcohol of approximately 18% was obtained from Acros Organics. N,N’-dimethylformamide (DMF) and hexamethylene diisocyanate (HDI) were purchased from Sigma-Aldrich, USA. Silver nanowires with 0.5 wt% dispersed in iso-propanol (IPA) were purchased from Nanopyxis, Korea (average diameter: 35 nm, length: 20 um). A poly(3,4-ethylenedioxythiophene)-poly(styrenesulfonate) (PEDOT:PSS) solution with a binding system was obtained from Heraeus Co., LTD (Clevios PH 1000).

### Preparation of scattering layers

The RNCs employed as scattering layers were fabricated using the RIE equipment. An RF power of 200 W was used for the SF_6_/O_2_ plasma treatment under a process pressure of 30 mTorr; 30 sccm of SF_6_ and 3 sccm of O_2_ were used for this treatment. The plasma treatment duration was varied to control the height and width of a high-aspect-ratio nanoscale corrugation. After etching by SF_6_/O_2_ plasma, high-density nanostructures with a high aspect ratio were fabricated. RNC of different heights and widths was fabricated by controlling the time of SF_6_/O_2_ plasma treatment. Because the amount of polymer etching was also affected by the power, flux of SF_6_/O_2_, and pressure, these values were fixed during the reactive ion etching. Other process conditions were fixed, and the time was changed. The SF_6_/O_2_ plasma treatment time of the RNC was 0, 300, 500 and 700 s.

### Fabrication of OLED device

After the RNC structure fabrication on the other side of the PVB/AgNW surface, the typical green phosphorescent OLEDs were deposited under a high-vacuum (10^−6^ torr) condition. The OLEDs consisted of 40 nm thick N,N′-bis(naphthalen-1-yl)-N,N′-bis(phenyl)- benzidine (NPB) and 10 nm thick 4,4′,4″-tris(carbazol-9-yl)triphenylamine (TCTA), which served as the hole transport layers, 30 nm thick 4,4′-bis(carbazol-9-yl)biphenyl (CBP) doped with 10 wt% Ir(ppy)3 (tris(2-phenylpyridine)iridium(III)) served as the phosphorescent green emitting layer, and 55 nm thick bis-4,6-(3,5-di-3-pyridylphenyl)-2-methylpyrimidine (B3PyMPM) was used as the electron transport layer. 0.4 nm thick LiF and 100 nm thick Al served as the cathode.

### Characterization

Scanning electron microscopy (FE-SEM, S-4800, Hitachi, Inc.) and atomic force microscopy (AFM, XE-100, Park System, Inc.) were used to measure the surface morphologies and profiles. UV-NIR spectrophotometer (Cary 5000, Agilent Technologies, Inc.) and spectroradiometer (pectra-Scan PR-670, Photo Research, Inc.) were used to measure the optical transmittances and EL characteristics in a dark box (≈0.1 lx, at room temperature) with a voltage sourcemeter (Model 237, Keithley Instruments, Inc.). A standard four-point probe system (resistivity meter, FPP-2400, Dasol ENG, Inc.) was used to measure the sheet resistance. A cyclic bending test was conducted using a bending machine (Z-tec, Inc.) with a digital multimeter for measuring the electrical resistance.

## Supplementary information

Supporting information
