# Supplementary material for: Extraction of Light Using Random Nanocone on Poly(vinyl-butyral) for Flexible OLEDs
Source: Sci Rep. 2019 Aug 23;9:12312. doi: 10.1038/s41598-019-48808-8 (PMC6707228; doi:10.1038/s41598-019-48808-8)
Supplement: Supplementary file 1 — Supporting information [file 41598_2019_48808_MOESM1_ESM.docx]

**Extraction of Light Using Random Nanocone on Poly(vinyl-butyral) for Flexible OLEDs**

**(Supplementary Information)**

**Dong Jun Lee^1,^** ^§^**, In Seon Yoon^1,^**^§^**,** [**Cheol Hwee Park**](https://onlinelibrary.wiley.com/action/doSearch?ContribAuthorStored=Park%2C+Cheol+Hwee)**^1^, Junhee Choi ^1^, Young Wook Park^2,*^, and Byeong-Kwon Ju^1,*^**

^§^ These authors contributed equally to this work.

^1^Display and Nanosystem Laboratory, School of Electrical Engineering, Korea University, 145, Anam-ro, Seongbuk-gu, Seoul 02841, Republic of Korea

^2^School of Mechanical and ICT Convergence Engineering, SUN MOON University, Chungcheongnam-do 31460, Republic of Korea

^*^Correspondence and requests for materials should be addressed to Y.W.P. (email: [zerook@sunmoon.ac.kr](mailto:zerook@sunmoon.ac.kr)) or B.-K.J. (email: bkju@korea.ac.kr)

Phone No.: +82-2-3290-3665

Fax. No.: +82-2-3290-3791

**Supplementary Information:**


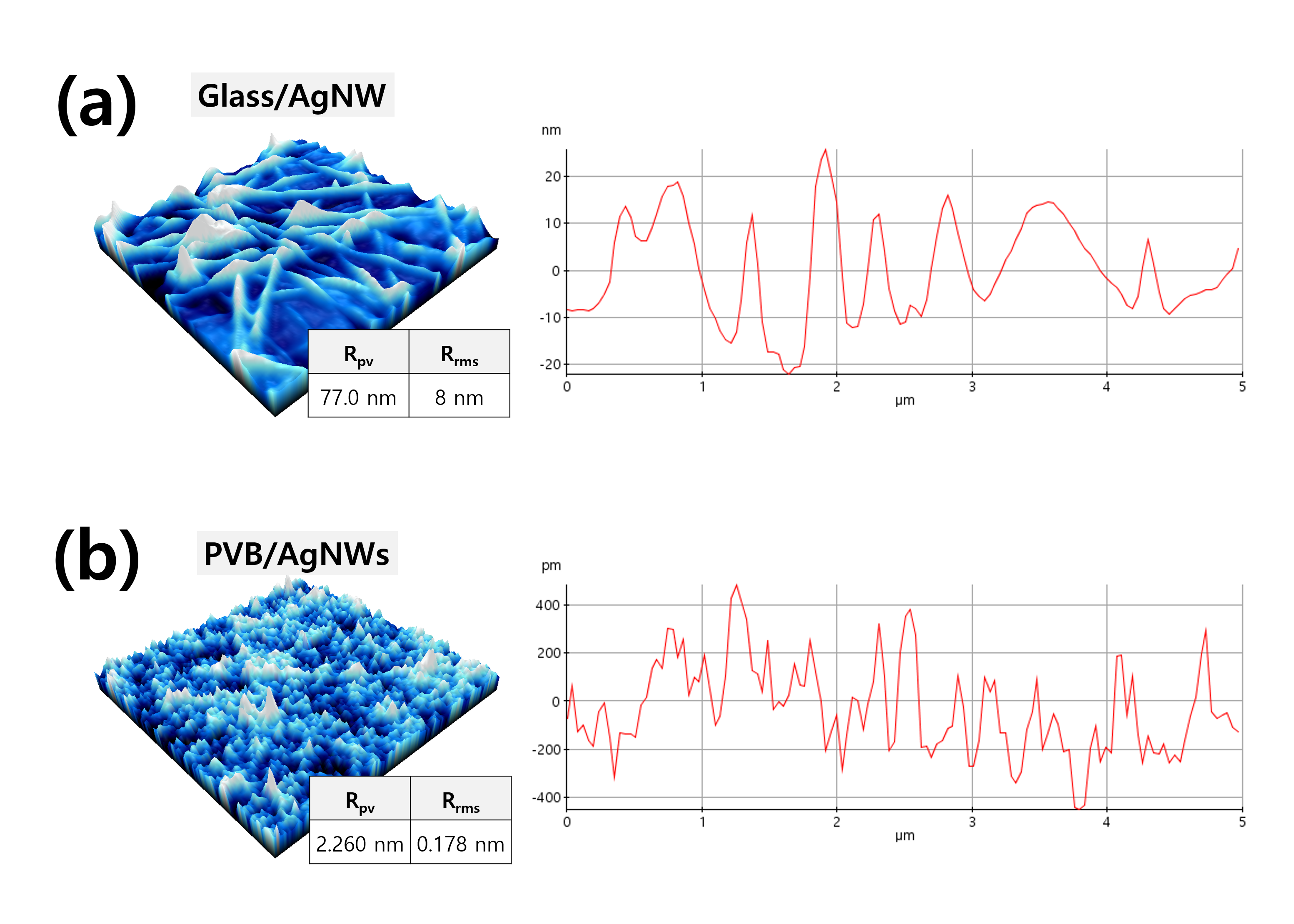


**Figure S1.** AFM topography images of (a) Glass/AgNW and (b) PVB/AgNW.


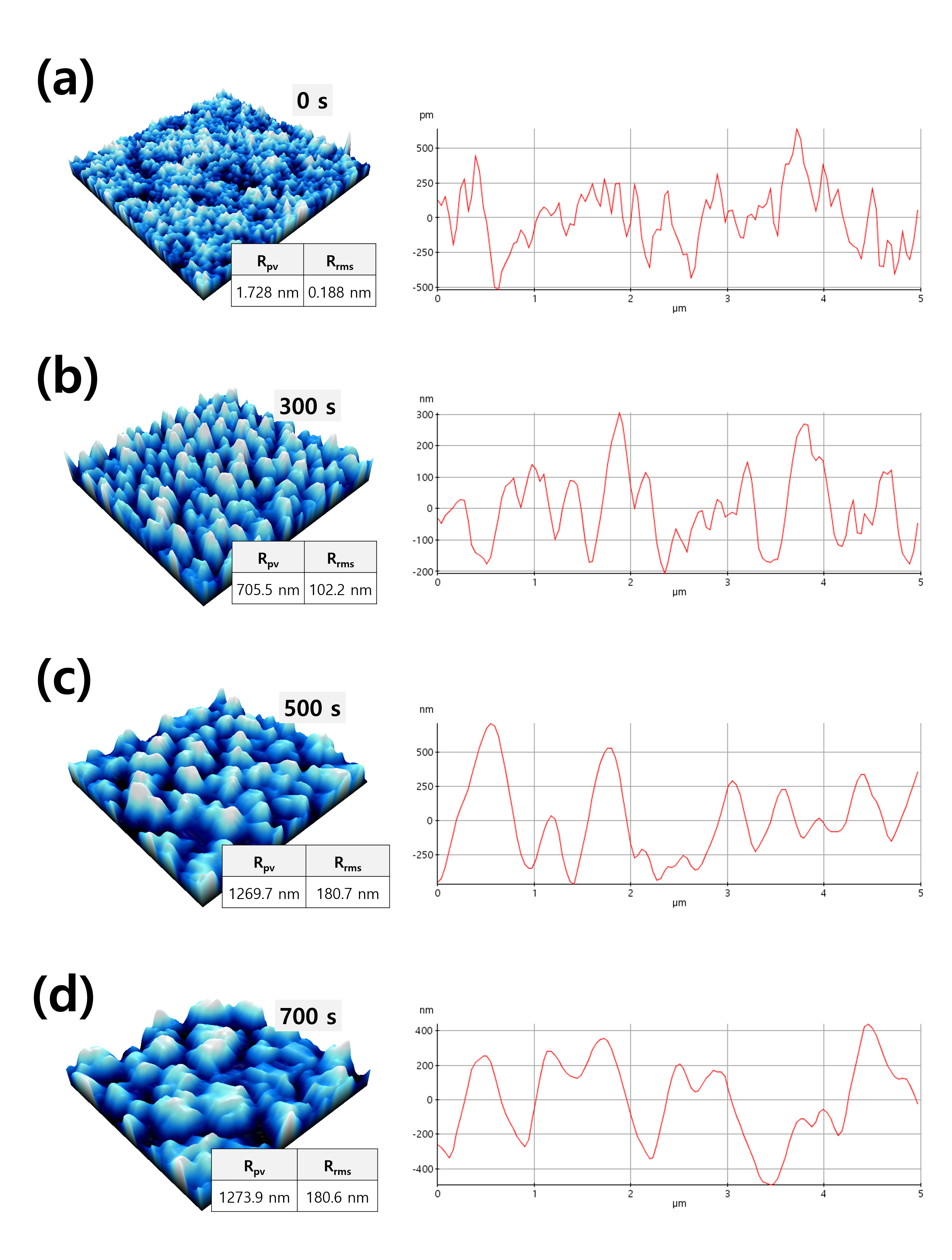


**Figure S2.** AFM images of plasma-treated PVB/AgNW substrate as a function of plasma time (process pressure: 30 mTorr, process power: 200 W, dimensions: 10 × 10 µm).


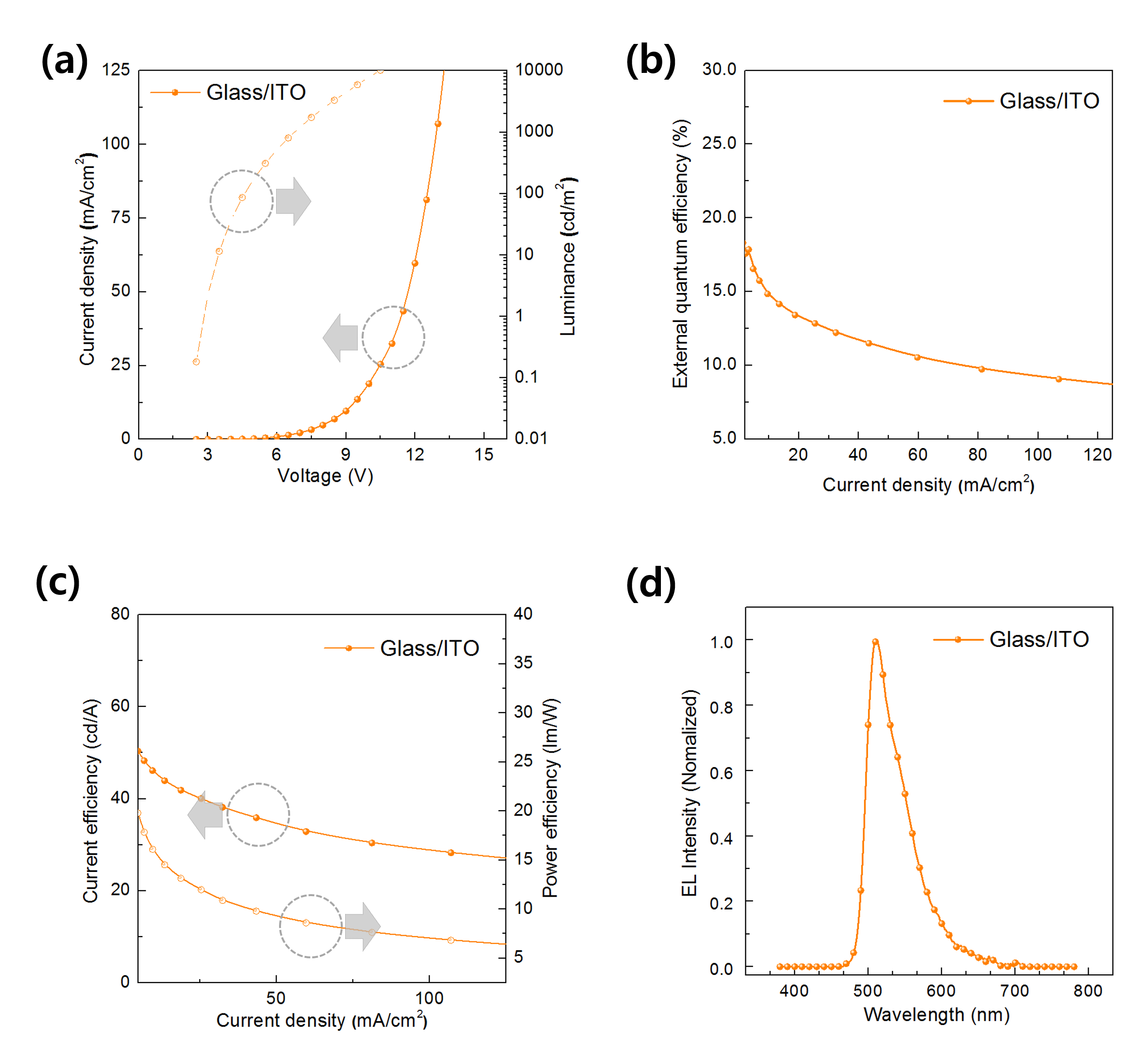


**Figure S3.** Performance characteristics of Glass/ITO green OLEDs. (a) Current density–luminance–voltage (J–L–V) characteristics of green OLEDs. (b) EQE as a function of current density. (c) CE and PE as a function of current density. (d) Normalized EL spectra in direction normal to glass substrate.
